# Traditional Chinese medicine constitution types and apolipoprotein B in hyperuricemia: Associations with cardiovascular risk

**DOI:** 10.5937/jomb0-57755

**Published:** 2025-11-05

**Authors:** Shuo Yang, Jinfeng Li, Hongli Zeng, Xueping Zhang, Deliang Xiong, Wenyi Tang, Bin Sun, Peng Yu, Xiaohong He, Weizheng Zhang

**Affiliations:** 1 Guangzhou University of Chinese Medicine, China; 2 The 11th People's Hospital of Guangzhou, Guangzhou Workstation of State Key Laboratory of Dampness Syndrome of Chinese Medicine, Guangzhou 510530 China; 3 The Second Affiliated Hospital of Guangzhou University of Chinese Medicine, Guangzhou 510105 China; 4 3The Second Affiliated Hospital of Guangzhou University of Chinese Medicine, Guangzhou 510105 China; 5 State Key Laboratory of Dampness Syndrome of Chinese Medicine (Guangdong Provincial Hospital of Chinese Medicine), Guangzhou 510006, China

**Keywords:** hyperuricemia, traditional Chinese medicine constitution, apolipoprotein B, homocysteine, cardiovascular risk, hiperurikemija, konstitucija tradicionalne kineske medicine, apolipoprotein B, homocistein, kardiovaskularni rizik

## Abstract

**Background:**

To explore the correlation between different traditional Chinese medicine (TCM) constitution types and apolipoprotein B (ApoB) in patients with hyperuricemia (HUA) and to investigate the relationships between TCM constitutions, uric acid levels, and various cardiovascular risk factors.

**Methods:**

A cross-sectional study involving 683 patients diagnosed with HUA was conducted. Patients' TCM constitutions were classified using the standardise "Classification and Determination of TCM Constitution" questionnaire. Serum uric acid (UA), lipid profiles, ApoB, and homocysteine (Hcy) levels were measured.

**Results:**

Among 683 HUA patients, phlegm-dampness (22.99% ) and damp-heat constitution (20.06% ) were the most common TCM constitution types. UA, ApoB, and Hcy levels in patients with phlegm-damp constitution were significantly higher than those in other constitutions (P&lt; 0.05). UA levels were negatively correlated with HDL-C (r=-0.472, P= 0.027) and positively correlated with ApoB (r= 0.618, P= 0.012) and Hcy (r= 0.492, P= 0.018).

**Conclusions:**

Phlegm-damp and damp-heat constitutions are the most common TCM constitution types in HUA patients and are associated with higher levels of UA, ApoB, and Hcy. These constitutional types are independently associated with increased cardiovascular risk.

## Abstract (sr)

**Uvod:**

Cilj je bio da se istraži povezanost između različitih tipova konstitucije prema tradicionalnoj kineskoj medicini (TCM) i nivoa apolipoproteina B (ApoB) kod pacijenata sa hiperurikemijom (HUA), kao i da se ispitaju odnosi između TCM konstitucije, nivoa mokracne kiseline i različitih faktora rizika za kardiovaskularne bolesti.

**Metode:**

Sprovedena je opservaciona studija preseka u koju je bilo uključeno 683 pacijenata sa dijagnozom HUA. Tipovi konstitucije su određeni pomocu standardizovanog upitnika "Klasifikacija i određivanje konstitucije prema TCM". Mereni su nivoi mokracne kiseline (UA), lipidni status, ApoB i homocistein (Hcy).

**Rezultati:**

Među 683 pacijenata sa HUA, najzastupljeniji tipovi konstitucije bili su flegmatično-vlažna (22,99% ) i vlažno-topla konstitucija (20,06% ). Nivoi UA, ApoB i Hcy kod pacijenata sa flegmatično-vlažnom konstitucijom su bili značajno viši nego kod ostalih tipova (P&lt; 0.05). Nivo UA bio je negativno korelisan sa HDL-C (r=-0,472, P= 0,027), a pozitivno sa ApoB (r= 0,618, P= 0,012) i Hcy (r= 0,492, P= 0,018).

**Zaključak:**

Flegmatično-vlažna i vlažno-topla konstitucija su najčešći tipovi prema TCM kod pacijenata sa HUA i povezane su sa višim nivoima UA, ApoB i Hcy. Ove konstitucije su nezavisno povezane sa povecanim kardiovaskularnim rizikom.

## Introduction

Hyperuricemia (HUA) refers to a class of metabolic diseases caused by the dysfunction of purine metabolism in the body of the patient due to some reason, which leads to an increase in blood uric acid levels or a decrease of blood uric acid excretion in the body. The patient is mainly manifested by an increase in blood uric acid level, which can only be found through the detection of blood biochemical indicators. Hyperuricemia itself doesn't usually cause symptoms [1] [2] [3]. In recent years, the incidence of HUA has increased in China, especially among elderly people and/or people in economically well-developed areas. Currently, studies have shown that the incidence of HUA shows a trend of low age. The development of HUA will not only cause patients to develop gouty arthritis and kidney disease but also be the main cause of hypertension and atherosclerosis. In addition, HUA is also related to the metabolism of blood glucose [4] [5].

The prevalence of HUA has been increasing globally, with recent estimates suggesting that it affects 21.4% of adults in the United States and 13.3% in China [6]. This rise in prevalence has been attributed to various factors, including changes in dietary patterns, increased obesity rates, and an ageing population [7]. The impact of HUA extends beyond its well-known association with gout, as emerging evidence suggests its role as an independent risk factor for cardiovascular diseases, chronic kidney disease, and metabolic syndrome [8].

Recent studies have highlighted the complex interplay between HUA and various metabolic and inflammatory pathways. Elevated uric acid levels have been shown to induce oxidative stress, activate the renin-angiotensin system, and promote endothelial dysfunction, all of which contribute to the development of cardiovascular complications [9]. Furthermore, the relationship between HUA and lipid metabolism has gained increasing attention, with studies demonstrating associations between uric acid levels and various lipid parameters, including apolipoprotein B (ApoB) [10].

There's increasing interest in complementary approaches like Traditional Chinese Medicine (TCM) for managing HUA alongside conventional uric acid-lowering therapies [11]. TCM's holistic approach offers a unique perspective on HUA through its concept of "constitution" or "body type" [12]. TCM constitution theory posits that individuals can be classified into different types based on their physical and psychological characteristics, which in turn influence their susceptibility to certain diseases and response to treatments [13]. This personalised approach aligns well with the current trend towards precision medicine and may offer valuable insights into the prevention and management of HUA and its associated complications [14].

However, the relationship between TCM constitution types and HUA, particularly in the context of lipid metabolism and cardiovascular risk factors, remains poorly understood. Previous studies have suggested potential associations between certain TCM constitution types and metabolic disorders, but comprehensive investigations specifically focusing on HUA are limited [10].

Therefore, this study aims to explore the correlation between different TCM constitution types and apolipoprotein B in patients with HUA, as well as investigate the relationships between TCM constitutions, uric acid levels, and various cardiovascular risk factors. By integrating TCM constitution theory with modern biochemical and clinical assessments, we hope to provide new insights into the pathophysiology of HUA and potentially identify novel strategies for its prevention and management.

## Materials and methods

### Study population

This cross-sectional study was conducted at the Guangzhou Municipal Health Management Center from June 2022 to July 2023. A total of 683 patients diagnosed with HUA were enrolled. The study was approved by the Ethics Committee of Guangzhou Municipal Health Management Center (Approval No. K2022-03), and all participants provided written informed consent.

Inclusion criteria: 1) Age ≥18 years; 2) Serum uric acid level >420 μmol/L (7.0 mg/dL) for men or >360 μmol/L (6.0 mg/dL) for women; 3) Willing to participate in the study and provide informed consent. Exclusion criteria: 1) Patients with mental disorders; 2) Patients who had taken steroid hormones within 2 weeks before enrollment; 3) Patients with malignant tumours; 4) Patients with secondary hyperuricemia (e.g., caused by hyperthyroidism, haematological disorders, or chemotherapy; 5) Pregnant or lactating women; 6) Patients with severe liver or kidney dysfunction.

### TCM constitution classification

The TCM constitution of each patient was determined using the »Classification and Determination of TCM Constitution« questionnaire developed by Wang Qi et al. [12]. This standardised questionnaire consists of 60 items covering various aspects of an individual's physical and mental characteristics. The questionnaire assesses nine constitution types: peaceful, qi-deficiency, yang-deficiency, yin-deficiency, phlegm-dampness, damp-heat, blood stasis, qi-stagnation, and special diathesis.

Patients completed the questionnaire under the guidance of trained TCM practitioners. The validity and reliability of the »Classification and Determination of TCM Constitution« questionnaire have been established in prior studies, demonstrating high internal consistency (Cronbach's =0.758-0.834 for subscales) and confirmatory factor analysis supporting its nine-factor structure (comparative fit index = 0.922, root mean square error of approximation = 0.06) [15]. Each item was scored on a 5-point Likert scale (1 = not at all, 2 = few, 3 = sometimes, 4 = often, 5 = always). The raw scores were converted to a percentage system. A constitution type was considered present if its score was 240 points. If multiple constitution types scored ≥40 points, the highest-scoring type was designated as the primary constitution.

### Clinical and biochemical measurements

### Anthropometric measurements: height and weight were measured with

Participants should wear light clothing and no shoes. Body mass index (BMI) was calculated as weight in kilograms divided by height in square meters. Waist circumference was measured at the midpoint between the lower border of the rib cage and the iliac crest. Blood pressure was measured in a seated position after 5 minutes of rest using a standard mercury sphygmomanometer.

### Blood sample collection and processing of fasting

Venous blood samples were collected from all participants in the morning after an overnight fast of at least 10 hours. Blood samples were collected in vacuum tubes containing EDTA for plasma separation and in tubes without anticoagulant for serum separation. Samples were centrifuged at 3000 rpm for 15 minutes at 4°C within 2 hours of collection. Aliquots of plasma and serum were stored at -80°C until analysis.

### Biochemical analyses

In this study, serum uric acid (UA), total cholesterol (TC), triglycerides (TG), high-density lipoprotein cholesterol (HDL-C), low-density lipoprotein cholesterol (LDL-C), fasting plasma glucose (FPG), and serum creatinine were measured using an automatic biochemical analyser (Hitachi 7600, Japan). At the same time, apolipoprotein B (ApoB) was determined using an immunoturbidimetric assay (Roche Diagnostics, Switzerland), plasma homocysteine (Hcy) was measured using an enzymatic cycling method (Roche Diagnostics, Switzerland), and glycosylated haemoglobin (HbA1c) was determined by high-performance liquid chromatography (Bio-Rad Variant II analyser).

### Assessment of cardiovascular diseases

Participants' medical histories were reviewed, and they underwent physical examinations, electrocardiograms, and carotid ultrasound examinations. Cardiovascular diseases included coronary heart disease (defined as a history of myocardial infarction, coronary revascularisation, or ≥50% stenosis in at least one major coronary artery), heart failure (diagnosed according to the European Society of Cardiology guidelines), and arrhythmias (confirmed by ECG or 24-hour Holter monitoring).

### Definition of cardiovascular risk factors

Hypertension was defined as a systolic blood pressure (SBP) ≥140 mmHg, a diastolic blood pressure (DBP) ≥90 mmHg, or the use of antihypertensive medications. Diabetes mellitus was diagnosed when fasting plasma glucose (FPG) ≥7.0 mmol/L, HbA1c ≥6.5%, or the use of antidiabetic medications was observed. Dyslipidemia was identified if total cholesterol (TC) ≥6.22 mmol/L, low-density lipoprotein cholesterol (LDL-C) ≥4.14 mmol/L, high-density lipoprotein cholesterol (HDL-C) <1.04 mmol/L, triglycerides (TG) ≥2.26 mmol/L, or the use of lipid-lowering medications was present. Obesity was classified as a body mass index (BMI) ≥28 kg/m^2^, according to Chinese criteria. Smoking status was determined as a current smoker or having quit smoking within the last year. Alcohol consumption was considered excessive if it exceeded 14 standard drinks per week for men or 7 for women.

### Statistical analysis

Statistical analyses were performed using SPSS version 25.0 (IBM Corp., Armonk, NY, USA). Continuous variables were expressed as mean±standard deviation (SD) for normally distributed data or median (interquartile range) for non-normally distributed data. Categorical variables were expressed as frequencies and percentages. The normality of data distribution was assessed using the Shapiro-Wilk test. For normally distributed data, one-way ANOVA was used for comparisons among multiple groups, followed by the LSD post-hoc test for pairwise comparisons. For non-normally distributed data, the Kruskal-Wallis test was applied, followed by Dunn's post-hoc test with Bonferroni correction to adjust for multiple comparisons. The chi-square test or Fisher's exact test was used to compare categorical variables between groups. Missing data (accounting for <2% of all variables) were handled using listwise deletion. Pearson correlation analysis was used to evaluate the relationships between UA and other biochemical parameters. Multiple linear regression analysis was performed to identify independent factors associated with serum UA levels. Multivariable logistic regression analysis was performed to assess the association between TCM constitution types and the presence of cardiovascular diseases, adjusting for age, gender, hypertension, diabetes, and smoking status. A two-tailed P-value <0.05 was considered statistically significant. Sensitivity analyses using the Holm-Bonferroni method confirmed the robustness of the primary findings.

### Sample size calculation

The sample size was calculated using G*Power 3.1.9.4 software. Based on a previous study [16] reporting a correlation coefficient of 0.3 between UA and ApoB, with a two-sided of 0.05 and a power of 0.90, the required sample size was calculated to be 112 participants. Considering potential dropouts and subgroup analyses, we aimed to recruit at least 500 participants.

## Results

### Demographic and clinical characteristics

A cohort of 683 HUA patients (mean age: 49.3 years; 66.6% male) was analysed. Key clinical characteristics included a mean BMI of 26.8 kg/m^2^ (indicating overweight status) and systolic/diastolic blood pressures of 133.4/82.8 mmHg, consistent with prehypertensive and hypertensive ranges (Table 1).

**Table 1:** Demographic and clinical characteristics of the study population. Data are presented as mean±SD or n (%).<br>Abbreviations: BMI, body mass index; HbA1c, glycated hemoglobin.

Characteristic	Characteristic
Age (years)	49.26±16.58
Gender (Male/Female)	455 (66.62%)/228 (33.38%)
BMI (kg/m^2^)	26.84±3.95
Waist circumference (cm)	92.15±10.73
Systolic blood pressure (mmHg)	133.42±17.86
Diastolic blood pressure (mmHg)	82.75±11.24
Fasting plasma glucose (mmol/L)	5.78±1.42
HbA1c (%)	5.92±0.87
Serum creatinine (mmol/L)	78.63±18.52

### TCM constitution distribution

Phlegm-dampness (23.0%) and damp-heat (20.1%) were the predominant TCM constitution types among HUA patients, collectively accounting for over 40% of cases. Less prevalent types included blood-stasis (14.1%) and yang-deficiency (11.1%), while peaceful constitution represented only 8.1% of the cohort (Table 2). This distribution underscores the predominance of dampness-related constitutions in HUA.

**Table 2:** Distribution of TCM constitution types. Abbreviations: TCM, traditional Chinese medicine.

TCM Constitution Type	n	Percentage (%)
Phlegm-dampness	157	22.99
Damp-Heat	137	20.06
Blood-stasis	96	14.06
Yang-deficiency	76	11.13
Idiosyncrasy	72	10.54
Peaceful	55	8.05
Yin-deficiency	38	5.56
Qi-stagnation	32	4.69
Qi-insufficiency	20	2.93
**Total**	**683**	**100**

### Gender-specific differences

Males exhibited significantly higher serum uric acid (UA: 455.5 vs. 381.4 μmol/L, P<0.001), apolipoprotein B (ApoB: 1.22 vs. 1.01 g/L, P=0.013), and homocysteine (Hcy: 15.2 vs. 13.6 μmol/L, P=0.007) compared to females. Conversely, HDL-C levels were lower in males (1.00 vs. 1.12 mmol/L, P=0.048), highlighting gender disparities in metabolic risk profiles (Table 3).

**Table 3:** Comparison of clinical data between genders. Data are presented as mean±SD. P-values <0.05 are considered statistically significant. Abbreviations: UA, uric acid; TC, total cholesterol; TG, triglycerides; HDL-C, high-density lipoprotein cholesterol; ApoB, apolipoprotein B; Hcy, homocysteine.

Parameter	Male<br>(n=455)	Female<br>(n = 228)	P-value
Age (years)	49.45±15.30	49.57±16.24	0.358
UA (mmol/L)	455.45±80.70	381.36±65.27	<0.001
TC (mmol/L)	4.90±0.89	4.86±0.64	0.157
TG (mmol/L)	1.70±1.36	1.69±1.32	0.214
HDL-C (mmol/L)	1.00±1.33	1.12±0.08	0.048
ApoB (g/L)	1.22±0.13	1.01±0.12	0.013
Hcy (mmol/L)	15.2±4.3	13.6±3.8	0.007

### TCM constitution and clinical parameters

Patients with phlegm-dampness constitution demonstrated markedly elevated UA (445.3 μmol/L), ApoB (1.42 g/L), and Hcy (16.8 μmol/L) compared to other constitutions (all P<0.05). For example, ApoB levels in phlegm-dampness patients were 32% higher than in damp-heat constitutions (1.42 vs. 1.08 g/L, P=0.004) and 35% higher than in peaceful constitutions (1.42 vs. 1.12 g/L, P=0.002) (Table 4).

**Table 4:** Comparison of clinical parameters among different TCM constitution types. Data are presented as mean±SD. *P <0.05 compared to other constitution types.<br>Abbreviations: UA, uric acid; ApoB, apolipoprotein B; Hcy, homocysteine.

TCM<br>Constitution<br>Type	UA<br>(mmol/L)	ApoB<br>(g/L)	Hcy<br>(mmol/L)
Phlegm-dampness	445.27±86.14*	1.42±0.19*	16.8±4.5*
Damp-Heat	425.13±72.25	1.08±0.13	14.6±3.9
Blood-stasis	425.26±72.36	1.07±0.11	14.2±4.1
Yang-deficiency	425.54±72.18	1.05±0.10	13.8±3.7
Idiosyncrasy	425.57±72.84	1.04±0.09	13.5±3.6
Peaceful	425.38±72.94	1.12±0.10	12.9±3.4
Yin-deficiency	425.41±72.05	1.00±0.11	13.7±3.8
Qi-stagnation	425.24±72.13	1.13±0.12	13.4±3.5
Qi-insufficiency	425.18±72.19	1.14±0.13	13.2±3.3
P-value	0.017	0.009	0.011

### Correlations with UA

UA levels showed a strong negative correlation with HDL-C (r=-0.472, P=0.027) and positive correlations with ApoB (r=0.618, P = 0.012), Hcy (r=0.492, P=0.018), BMI (r=0.385), waist circumference (r=0.412), and systolic blood pressure (r=0.328) (all P<0.05). These correlations suggest a synergistic interplay between hyperuricemia, dyslipidemia, and adiposity (Table 5).

**Table 5:** Correlation between UA levels and other biochemical parameters. Abbreviations: BMI, body mass index; HDL-C, high-density lipoprotein cholesterol; ApoB, apolipoprotein B; Hcy, homocysteine.

Parameter	Correlation<br>Coefficient (r)	P-value
HDL-C	-0.472	0.027
ApoB	0.618	0.012
Hcy	0.492	0.018
BMI	0.385	0.031
Waist circumference	0.412	0.024
Systolic blood pressure	0.328	0.039
Fasting plasma glucose	0.297	0.045

### Predictors of UA levels

Multiple linear regression revealed that male gender (β=0.312, P<0.001), BMI (β=0.245, P= 0.002), ApoB (β=0.287, P<0.001), Hcy (β=0.176, P = 0.015), and phlegm-dampness constitution (β=0.223, P=0.003) independently predicted elevated UA levels, collectively explaining 58% of the variance (adjusted R^2^=0.58) (Table 6).

**Table 6:** Multiple linear regression analysis for factors associated with serum UA levels. Abbreviations: BMI, body mass index; ApoB, apolipoprotein B; Hcy, homocysteine.

Variable	β Coefficient	95% CI	P-value
Male gender	0.312	0.186-0.438	<0.001
BMI	0.245	0.128-0.362	0.002
ApoB	0.287	0.159-0.415	<0.001
Hcy	0.176	0.063-0.289	0.015
Phlegm-dampness<br>constitution	0.223	0.104-0.342	0.003

### Cardiovascular disease (CVD) incidence

High ApoB levels were associated with a nearly doubled CVD incidence in males (42.7% vs. 22.8%, P<0.001) and a 50% increase in females (37.8% vs. 25.2%, P=0.036). Stratified analysis showed that males with the phlegm-dampness constitution and high ApoB had the highest CVD risk (51.2%) (Table 7).

**Table 7:** Incidence of Cardiovascular Diseases by ApoB Levels and Gender. Abbreviations: CVD, cardiovascular diseases; ApoB, apolipoprotein.

Gender	ApoB<br>Level	n	CVD<br>Incidence	P-value
Male	High	227	97 (42.73%)	<0.001
Low	228	52 (22.81%)		
Female	High	119	45 (37.82%)	0.036
Low	119	30 (25.21%)		

### CVD risk factors

Logistic regression identified phlegm-dampness (OR=2.18, 95% CI:1.42-3.35) and damp-heat constitutions (OR=1.86, 95% CI : 1.21—2.87) as independent predictors of CVD, surpassing traditional risk factors like hypertension (OR=1.68) and diabetes (OR=1.53). Notably, high ApoB conferred a 95% increased risk (OR = 1.95, 95% CI : 1.33-2.86), emphasising its role in HUA-related cardiovascular pathology (Table 8).

**Table 8:** Logistic regression analysis for cardiovascular diseases. Abbreviations: ApoB, apolipoprotein.

Variable	Odds Ratio	95% CI	P-value
Phlegm-dampness<br>constitution	2.18	1.42-3.35	<0.001
Damp-Heat constitution	1.86	1.21-2.87	0.005
High ApoB level	1.95	1.33-2.86	0.001
Age (per 10 years)	1.42	1.18-1.71	<0.001
Male gender	1.37	0.94-2.01	0.102
Hypertension	1.68	1.15-2.45	0.007
Diabetes	1.53	1.04-2.25	0.031
Smoking	1.45	0.98-2.14	0.062

## Discussion

This study provides a comprehensive analysis of the relationships between TCM constitution types, HUA, and cardiovascular risk factors in a large cohort of patients. Our findings offer novel insights into the complex interplay between TCM constitutional theory and modern biomedical parameters in the context of HUA and associated cardiovascular complications.

Our study revealed that phlegm-dampness (22.99%) and damp-heat (20.06%) were the most prevalent TCM constitution types among HUA patients. This finding is consistent with previous studies on metabolic disorders. It supports the TCM theory that these constitution types are prone to the accumulation of dampness and heat in the body, which may contribute to the development of HUA [17]. The high prevalence of these constitution types in our HUA cohort suggests that TCM constitutional assessment may provide valuable information for identifying individuals at higher risk of developing HUA.

The significant differences observed between male and female HUA patients in terms of UA levels, HDL-C, ApoB, and Hcy highlight the importance of considering gender-specific factors in HUA management. The higher UA levels in males are consistent with previous epidemiological studies and can be attributed to the uricosuric effect of estrogen in females [18]. The gender differences in lipid profiles and Hcy levels suggest that the pathophysiological mechanisms and cardiovascular risk associated with HUA may vary between males and females, warranting tailored prevention and treatment strategies.

Our results demonstrated that patients with phlegm-dampness constitution had significantly higher levels of UA, ApoB, and Hcy compared to other constitution types. This finding supports the TCM concept that phlegm-dampness constitution is associated with metabolic imbalances and a pro-inflammatory state [19]. The elevated levels of these biomarkers in phlegm-dampness constitution patients suggest a potential mechanistic link between this TCM constitution type and the development of HUA and associated cardiovascular risk.

The significant correlations observed between UA levels and various cardiovascular risk factors, including ApoB, Hcy, BMI, and blood pressure, underscore the complex relationship between HUA and cardiovascular health. These findings are in line with growing evidence suggesting that HUA may contribute to cardiovascular risk through multiple pathways, including lipid metabolism dysregulation, chronic inflammation, and endothelial dysfunction [20].

The logistic regression analysis revealed that phlegm-dampness and damp-heat constitutions were independently associated with increased risk of cardiovascular diseases, even after adjusting for traditional risk factors. This novel finding suggests that TCM constitution assessment may provide additional prognostic information beyond conventional risk factors. The integration of TCM constitutional theory into cardiovascular risk assessment could potentially enhance risk stratification and guide personalised prevention strategies [21].

Our study demonstrated a strong positive correlation between UA and ApoB levels, and patients with high ApoB levels had a significantly higher incidence of cardiovascular diseases. These findings support the emerging role of ApoB as a valuable marker of cardiovascular risk in HUA patients [22]. The association between ApoB and cardiovascular outcomes in our cohort suggests that lipid-lowering strategies targeting ApoB may be particularly beneficial in HUA patients, especially those with phlegm-dampness constitution.

The identification of specific TCM constitution types associated with HUA and increased cardiovascular risk has important implications for clinical practice. Patients with phlegm-dampness and damp-heat constitutions may benefit from more intensive monitoring and earlier interventions to prevent the development of HUA and its complications. TCM treatments tailored to these constitution types, such as herbs that promote fluid metabolism and reduce inflammation, could be explored as complementary approaches to conventional HUA management [23].

The phlegm-dampness constitution, characterised by obesity, lipid dysregulation, and chronic inflammation, shares striking parallels with metabolic syndrome in Western medicine, which similarly encompasses central obesity, insulin resistance, and dyslipidemia [24]. This overlap suggests that TCM's emphasis on resolving »dampness« may align with targeting metabolic dysfunction. Our findings further indicate that elevated ApoB, a key atherogenic lipoprotein, is strongly linked to cardiovascular risk in HUA patients. Mechanistically, ApoB-rich lipoproteins promote endothelial dysfunction and plaque formation while stimulating inflammatory pathways. Elevated ApoB levels correlate with increased IL-6 and CRP [25], markers of systemic inflammation that exacerbate vascular injury. In phlegm-dampness patients, the synergy between hyperuricemia, lipid abnormalities, and inflammation likely amplifies cardiovascular risk [26]. These insights bridge TCM constitutional theory with Western pathophysiology, underscoring the potential of combined approaches, such as anti-inflammatory therapies alongside dampness-resolving herbs, to mitigate HUA-related complications.

The strengths of this study include its large sample size, comprehensive assessment of both TCM constitution types and modern biomedical parameters and the novel integration of TCM theory with cardiovascular risk assessment in HUA patients. However, several limitations should be acknowledged. First, the cross-sectional design precludes the establishment of causal relationships between TCM constitution types, HUA, and cardiovascular outcomes. Longitudinal studies are needed to elucidate the temporal relationships and predictive value of TCM constitution types in HUA progression and cardiovascular risk. Second, while we adjusted for several confounding factors in our analyses, residual confounding cannot be ruled out. Third, the study was conducted in a single centre, which may limit the generalizability of our findings to other populations.

Future research should focus on validating our findings in diverse populations and exploring the underlying mechanisms linking TCM constitution types to HUA and cardiovascular risk. Prospective studies are needed to evaluate the long-term outcomes associated with different TCM constitution types in HUA patients. Additionally, interventional studies investigating the efficacy of TCM constitution-based treatments in managing HUA and reducing cardiovascular risk would be valuable. The integration of genomic and metabolomic approaches with TCM constitutional theory could potentially uncover novel biomarkers and therapeutic targets for personalised HUA management.

In conclusion, our study provides compelling evidence for the association between specific TCM constitution types, particularly phlegm-dampness, and increased cardiovascular risk in HUA patients. The integration of TCM constitutional assessment with conventional risk factors and biomarkers such as ApoB may enhance our ability to identify high-risk individuals and tailor prevention and treatment strategies. Clinically, patients identified with phlegm-dampness or damp-heat constitutions could benefit from early lifestyle interventions (e.g., dietary modifications to reduce dampness-inducing foods) and targeted TCM therapies (e.g., herbs like Coicis Semen or Atractylodis Rhizoma to resolve dampness). Additionally, integrating TCM constitution screening into routine cardiovascular risk assessments could enable personalised management plans, such as intensified lipid-lowering regimens for high-ApoB individuals with phlegm-dampness traits. These findings highlight the potential value of incorporating TCM concepts into modern medical practice for a more holistic and personalised approach to HUA management and cardiovascular disease prevention.

## Dodatak

### Funding

This work was supported by The Guangzhou Science and Technology Plan Project (No. 2024A03J0105) and the Guangdong Provincial Administration of Traditional Chinese Medicine (No. 20231138).

### Authors' contribution

Shuo Yang, Jinfeng Li, Xiaohong He, and Weizheng Zhang contributed to this work equally.

### Conflict of interest statement

All the authors declare that they have no conflict of interest in this work.
